# Chronic Aerobic Exercise Decreases Lectin-Like Low Density Lipoprotein (LOX-1) Receptor Expression in Heart of Diabetic Rat

**DOI:** 10.7508/ibj.2016.01.004

**Published:** 2016-01

**Authors:** Simin Riahi, Mohammad Taghi Mohammadi, Vahid Sobhani, Shima Ababzadeh

**Affiliations:** 1 Exercise Physiology Research Center, Baqiyatallah University of Medical Sciences, Tehran, Iran;; 2 Dept. of Physiology and Biophysics, Faculty of Medicine, Baqiyatallah University of Medical Sciences, Tehran, Iran;; 3 Dept. Anatomy, Faculty of Medicine, Qom University of Medical Sciences, Qom, Iran

**Keywords:** Hyperglycemia, Diabetic complications, LOX-1 receptor, Exercise, Free radicals

## Abstract

**Background:**

Overexpression of lectin-like low density lipoprotein (LOX-1) receptor plays an important role in hyperglycemia-induced vascular complications such as atherosclerosis. Based on the beneficial effects of exercise on preventing cardiovascular complications of diabetes, we aimed to examine the protective effects of aerobic exercise on expression of LOX-1 receptor and production of free radicals in the heart of diabetic rats.

**Methods:**

Four groups of rats were used: (n = 5 per group): sedentary normal, trained normal, sedentary diabetes and trained diabetes. Diabetes was induced by a single intraperitoneal injection of streptozotocin (50 mg/kg). The exercise protocol was consisted of swimming 30 min/day, 5 days/week for eight weeks. Plasma glucose was evaluated at initiation, weeks 4 and 8 of experiment. At the end of experiment, rats were sacrificed and the heart was removed for determination of nitrate, malondialdehyde, and LOX-1 gene expression.

**Results:**

In normal non-diabetic rats, the blood glucose level was <150 mg/dl; however, the induction of diabetes resulted in levels more than >400 mg/dl. Gene expression of LOX-1 was increased in the heart of diabetic rats. Exercise reduced the gene expression of this protein in diabetic states without reducing the blood glucose. Finally, swimming exercise decreased the malondialdehyde and nitrate levels in heart tissue both in control and diabetic rats.

**Conclusion:**

Swimming exercise reduces heart expression of the LOX-1 receptor in accompany with reduction of free radicals production. Since these parameters are important in generation of diabetic complications, swimming exercise is a good candidate for reducing these complications.

## INTRODUCTION

Diabetes mellitus is a common chronic metabolic disease that results in deregulation of serum glucose and hyperglycemia. One of the most important complications of chronic hyperglycemia is cardiovascular diseases (CVD), which leads to early morbidity and mortality^[^^1^^]^. This type of death in diabetic patients is 2-5-fold more than non-diabetic patients. The exact mechanism of diabetes causing CVD is not clear but some evidence shows that the level and duration of hyperglycemia may correlate to the incidence of CVD complications^[^^2^^]^. Overproduction of different free radicals and oxidative stress is the main factor that links diabetes to CVD. Prolong hyperglycemia by excessive production of reactive oxygen species (ROS) and weakening antioxidant defense system results in heart oxidative stress^[^^3^^]^. One of the most important toxic effects of ROS is the production of oxidize low-density lipoprotein (oxLDL)^[^^4^^]^. OxLDL contributes to CVD complications through activation of lectin-like low density lipoprotein (LOX-1) receptor, which is overexpressed during chronic hyperglycemia^[^^4^^]^. Binding of different ligands to LOX-1 receptor activates some intracellular pathways that are involved in inflammatory responses and lipid deposition in vulnerable vessels to atherosclerosis such as heart coronary arteries^[^^5^^]^.

Oxidative stress due to hyperglycemia is the main risk factor that mediates overexpression of LOX-1 receptor in diabetic condition^[^^6^^]^. Based on some recent studies, this receptor contributes to inflammatory responses, apoptosis, and athrogenic effects through the activation of nuclear factor-KB^[^^6^^,^^7^^]^. It is appeared that the inhibition of this receptor is a useful way for prevention of CVD complications of diabetes such as atherosclerosis^[^^8^^]^. Exercise training, diet, and medication are three main cornerstones in management of diabetes. Researchers have demonstrated that regular exercise prevents many complications of type II diabetes^[^^9^^,^^10^^]^. Evidence shows that people who have active lifestyle are less susceptible to insulin resistance and impaired glucose tolerance^[^^10^^]^. Epidemiological findings demonstrate some beneficial effects of physical activity on the reduction of CVD in diabetic patients^[^^11^^]^. Regular moderate-intensity exercise also reduces the mortality rate due to long-suffering CVD^[^^12^^]^. The study of Motoyuki e*t al*.^[^^13^^]^ indicated that the prevalence of atherosclerosis in people with low cardiopulmonary fitness is more than individuals with high cardiopulmonary fitness. Also, the study of Ramachandran *et al.*^[^^14^^]^ demonstrated that moderate aerobic exercise reduces the size of preexisting atherosclerotic lesions in LDL receptor knockout mice fed with high fat diet. Although reduction in the risk of cardiovascular mortality and morbidity due to exercise is obvious, the exact mechanisms that exercise prevents atherosclerosis formation is unknown. Therefore, the present study was performed to reveal some beneficial effects of swimming exercise on CVD through alterations of LOX-1 receptor expression and changing free radicals production in the heart of diabetic rat.

## MATERIALS AND METHODS

**Animals**

Male wistar rats (200 ± 20 g) were purchased from Pasture Institute of Iran (Tehran). During the experiment, all animals were kept in standard polyester cages (two rats in each cage) in a room with standard temperature (22 ± 2ºC) and humidity (55 ± 5%) with 12 h light/dark cycle and free access to water and standard rodent chow. All protocols of the study were approved by Institutional Animal Ethics Committee of Baqiyatallah University of Medical Sciences (Iran), which followed the NIH guidelines for care and use of animals.

**Induction of diabetes**

Diabetes was induced by a single intraperitoneal injection of 50 mg/kg streptozotocin (Sigma, USA) dissolved in citrate buffer (0.01 mol/L, pH 4.5) while the non-diabetic rats received citrate buffer solution in the same volume. Five days later, blood samples obtained from retro-orbital plexuses vein were used for monitoring serum glucose. The rats included diabetes when the serum glucose concentration was more than 400 mg/dl, and those with serum glucose less than 400 mg/dl were excluded from the study^[^^15^^]^. 

**Experimental groups and design**

Rats were randomly divided into four groups (n = 5 per group): Normal rats (sedentary normal) were healthy animals that remained sedentary, trained normal group were healthy animals that did exercise for eight weeks, sedentary diabetic group were diabetic animals that remained sedentary, and trained diabetic group were diabetic animal that did exercise for eight weeks. Blood samples were collected from retro-orbital plexuses (4 and 8 weeks after the beginning of exercise) under light anesthesia with ether. Serum was separated by centrifugation at 3500 ×g for 15 min, and the concentration of serum glucose was determined by using the available commercial kit (ZiestChemie Diagnostic Co., Iran). The rats were sacrificed 48 hour after the last training session and hearts were removed. After washing with cold phosphate buffer saline and snap freezing in liquid nitrogen, the hearts were stored in -80ºC for evaluation of gene expression and measurement of nitrate (NOx) and malondialdehyde (MDA) levels.

**Exercise protocol**

In the present study, we used endurance swimming as a model of exercise intervention. The training included daily moderate-intensity swimming for eight weeks, which can induce cardiac hypertrophy^[^^16^^]^. The rats in swimming groups performed swimming in a rubber swimming tank with dimension of 55 _×_ 100 _×_ 60 cm for 30 min in the morning. The water depth was enough to prevent from resting and eliminate bobbing behavior. The tank was filled with tap water that was sufficient for six rats to swim simultaneously. Water temperature was fixed at 32 ± 2ºC to prevent hypothermia. The exercise program in the first week of training was begun with acclimatization to water. In the first day, rats swam for 10 min. Then duration of training was increased 10 min daily until each rat could swim continuously for 30 min. In subsequent weeks, the rats could swim 30 min a day for five times a week (30 min/day; 9:00-11:00 AM on Saturday to Wednesday)^[^^17^^]^. The control groups (normal and diabetes) were remained sedentary in the swimming tank while it was filled with tap water in 5 cm depth that animal’s paws reached to the bottom of tank. After each session, the animals were dried and kept in a warm place to prevent from hypothermia stress.

**Evaluation of gene expression**

Gene expression of LOX-1 receptor was determined using semi-quantitative reverse transcriptase-polymerase chain reaction (RT-PCR). Total RNA was extracted from 50 mg of heart tissue using the RNA extraction kit (Topagene Kavosh, Iran) according to the manufacturer’s protocol. The quantity and quality of the extracted RNA samples were estimated by spectrophotometry at 260 and 280 nm. Complementary DNA (cDNA) was synthesized from 5 μg total RNA using the Revert Aid First Strand cDNA Synthesis Kit (BIONEER, Korea). Expression of the β-actin housekeeping gene was used as the reference for the level of target gene expression. cDNA (2 µl) was amplified with PCR kit (BIONEER, Korea) according to the manufacturer’s protocol. Also, appropriate primers were used for LOX-1 (forward: TTTAGGACCAGGGGCGTTTC and reverse: GGAG ATGGACCCAAGTCGTG) and β-Actin (forward: CCACACCCGCCACCAGTTCG and reverse: CTA GGGCGGCCCACGATGGA) genes. The products of PCR-amplified samples were visualized on 1.5% agarose gel using ethidium bromide. The gel images were digitized by using the Gel Doc (Kiagene, Iran), and the images of the stained sections were also taken^[^^18^^]^.

**Evaluation of oxidative and nitrosative stress**

The fractions of thawed tissue samples were weighed, and added to homogenization medium (phosphate buffer, 0.1 mol, pH 7.4). After homogenizing of tissues on ice by an electric homogenizer, the samples were centrifuged (at 4ºC, 4500 ×g, for 20 minute), and supernatant was removed as the heart cytosolic extract and stored in -80ºC for analysis of NOx and MDA levels. 

**Nitrate** **assay**

The NOx in the ‘cytosolic extract’ was measured by the colorimetric reaction of the Griess reagent. In brief, 0.1 ml cytosolic extract was deproteinized by adding 0.2 ml zinc sulfate solution and centrifuged at 4ºC at 4500 ×g for 20 minute to separate supernatant for NOx assay. Afterwards, 0.1 ml supernatant (as sample) or pure water (as blank) or sodium nitrate (as standard) was mixed with 0.1 ml vanadium III chloride to reduce nitrate to nitrite. Next, 0.05 ml sulfanilamide (0.01 %) and 0.05 ml N-[1-naphthyl] ethylenediamin dihydro-chloride (0.01%) were incubated in dark place at 37ºC for 30 minute. Finally, the absorbance of solution was determined at a wave length of 540 nm. Nitrite concentration was estimated from a standard curve generated from the absorbance of each sodium nitrate solution^[^^19^^]^, and the nitrite-nitrate levels were expressed as nmol/mg protein.

**Malondialdehyde assay**

The amount of lipid peroxidation was measured by the assessment of MDA formation using thiobarbituric acid assay^[^^20^^]^. Trichloroacetic acid (2.5 ml, 20%), sulfuric acid (2.5 ml, 0.05 M), and thiobarbituric acid (3 ml, 0.2 g/dl) were added to cytosolic extract (500 μl) and then vortexed. The mixture was placed in a water bath at 95ºC for 30 min. After cooling in runny water, 2 ml n-butanol was added and vigorously vortexed. Following centrifugation, the absorbance of the upper pink color phase was determined at 532 nm. Finally, tetraethoxypropane was used to prepare a standard curve, and results were expressed as nmol/mg protein. Protein content in the cytosolic extracts was determined spectrophotometrically with Bradford's method at wave length of 595 nm^[^^20^^]^. Bovine serum albumin was used as the standard protein. The concentration of protein was expressed as mg/ml, and the amount of MDA and NOx in each sample was normalized to the cytosolic protein concentration. Finally, the results were expressed as nmol/mg of the cytosolic protein (nmol/mg protein).

**Statistical analyses**

The results were expressed as the means ± SEM. All statistical comparisons were carried out using one-way analysis of variance (ANOVA) and Tukey's test as post-hoc analysis. *P*<0.05 was considered statistically significant. 

## RESULTS

**Plasma glucose**

The concentration of plasma glucose for different groups of our experiment at the first day and the end of weeks 4 and 8 is shown in Table 1. The mean value of glucose for these times was <150 mg/dl in normal sedentary rats. Swimming exercise had not significant effects on plasma glucose of trained normal rats. Induction of diabetes significantly increased the concentration of plasma glucose (above 450 mg/dl). Finally, swimming exercise did not change the plasma glucose of trained exercise rats. 

**Gene expression of LOX-1 protein**

**Fig. 1.**
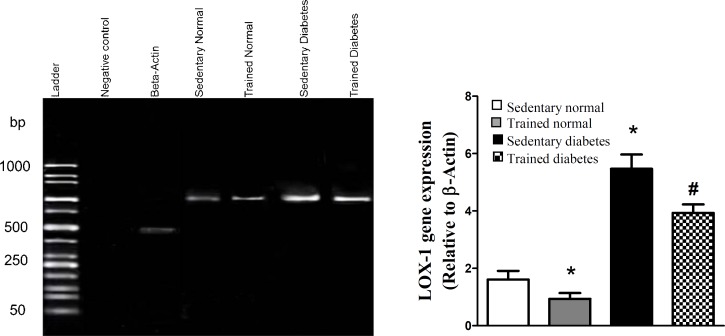
mRNA of lectin-like oxidized low-density lipoprotein (LOX-1) receptor in cardiac cells of different experimental groups (left hand). RT-PCR products were visualized on agarose gel (1.5%) using ethidium bromide staining method. The graph shows the density of LOX-1 gene that was normalized relative to density of β-Actin gene for different groups (right hand). All values are presented as mean ± SEM. *Significantly different from sedentary normal group (*P*<0.05); ^#^Significantly different from sedentary diabetic group (*P*<0.05

Figure 1 shows the LOX-1 gene expression of heart cells in different groups of our experiment. Based on formed band, the gene of LOX-1 protein was expressed in heart cells of sedentary normal rats. Exercise in normal rats attenuated the gene expression of LOX-1 receptor. On the other hands, the induction of diabetes induced overexpression of this gene in heart of sedentary diabetic group; however, swimming exercise reduced the gene expression of this protein in heart of trained diabetic rats.

**Table 1:** Representative changes of blood glucose (mg/dl) in sedentary (normal and diabetic groups) and exercised (trained normal and trained diabetes) rats at the beginning (day 1), after 4, and 8 weeks of swimming exercise

**Groups**		**Glucose (mg/dl)**	
	**day 1**	** week 4**	** week 8**
Sedentary normal	117 ± 4	115 ± 3	133 ± 7
Trained normal	119 ± 5	123 ± 4	111 ± 8
Sedentary diabetes	530 ± 70*	497 ± 29*	515 ± 26*
Trained diabetes	537 ± 43*	496 ± 23*	461 ± 42*

All values are presented as mean ± SEM.

* Significantly different from sedentary normal group (*P*<0.05).

**Parameters of oxidative and nitrosative stress**

**Fig. 2:**
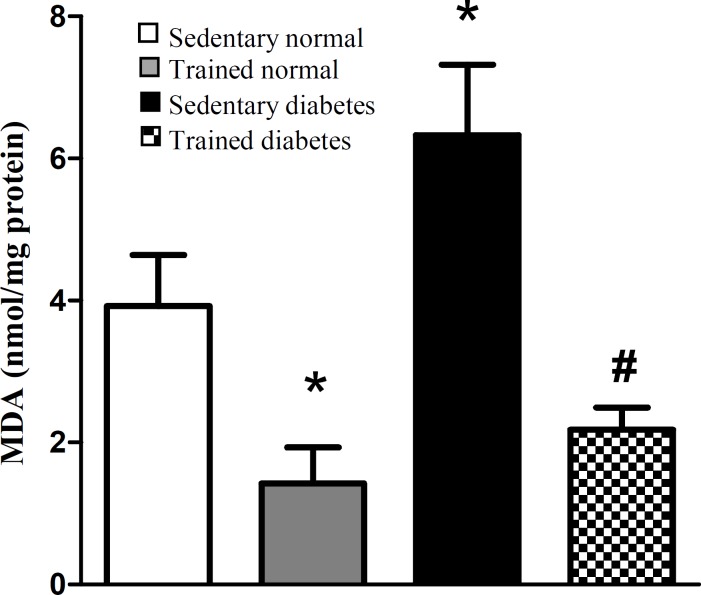
Effect of eight-week swimming exercise on malondialdehyde (MDA) content in cardiac tissue. MDA values are indicated as nmol/mg protein. All values are presented as mean ± SEM. *Significantly different from sedentary normal group (*P*<0.05); ^#^Significantly different from sedentary diabetic group (*P*<0.001

**Fig. 3:**
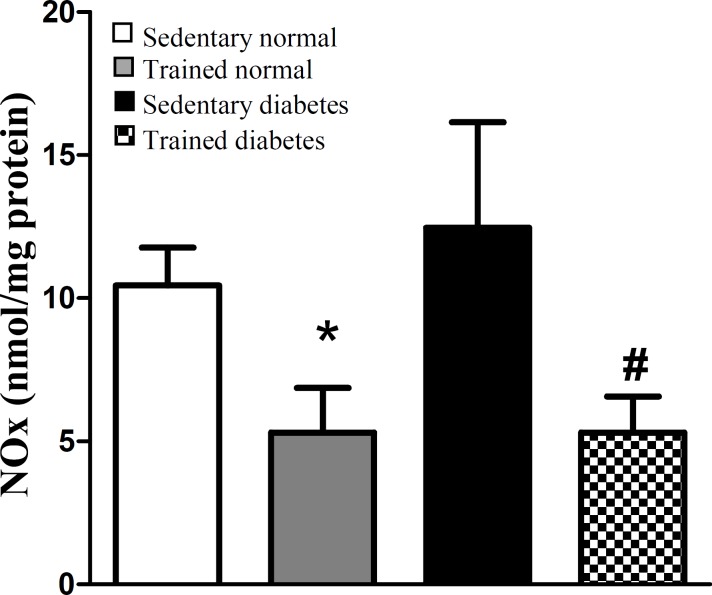
Effect of eight-week swimming exercise on nitrate (NOx) content in cardiac tissue. NOx values are indicated as nmol/mg protein. All values are presented as mean ± SEM. *Significantly different from sedentary normal group (*P*<0.05); ^#^Significantly different from sedentary diabetic group (*P*<0.01

Figure 2 shows the MDA content of heart tissue, as the index of oxidative stress, in different experimental groups after eight weeks. Induction of diabetes significantly increased the MDA content of heart tissue, whereas swimming exercise significantly attenuated the MDA content of heart tissue in trained diabetic group compared with sedentary diabetic rats. Also, swimming exercise significantly could reduce the MDA content of heart tissue in trained normal rats. Total NOx (nitrite and nitrate) content of heart tissue, as the index of nitrosative stress, was significantly decreased in trained normal animals after eight weeks of swimming exercise. Also, swimming exercise significantly could reduce the NOx content of heart tissue in trained diabetic rats (Fig. 3).

## DISCUSSION

In the present study, we showed some beneficial effects of swimming exercise on diabetic condition via decrement of LOX-1 receptor. The findings of the current study indicated that swimming exercise attenuates the gene expression of LOX-1 protein in diabetic condition (Fig. 1). On the other hands, this type of exercise decreased the parameters of oxidative and nitrosative stress in heart tissue following diabetic condition (Figs. 2 and 3). Based on our findings, it is appeared that swimming exercise is able to reduce oxidative and nitrosative stress of heart tissue possibly via reduction of LOX-1 receptor expression. 

Our results showed that the gene expression of LOX-1 receptor was increased in heart tissue following diabetes (Fig. 1). Therefore, the present findings emphasize the role of hyperglycemia in overexpression of this receptor.

The study of Li *et al.*^[^^21^^]^ indicated that high blood glucose has a potent effect on the LOX-1 expression through increment of free radicals production. The expression of this protein has been demonstrated in different cells of atherosclerotic plaque formation sites such as endothelial and vascular smooth muscle cells in aorta,carotid, and coronary arteries^[^^22^^]^. Also, some other different cells such as cardiomyocytes, macrophages, and platelets express the LOX-1 receptor in hyperglycemic situations^[^^22^^]^. According to the study of Nagase *et al.*^[^^23^^]^, oxidative injury to endothelium of arteries is the main factor in initiation and development of atherosclerosis. This can be due to upregulation of LOX-1 protein in these susceptible places. In other words, LOX-1 receptor is a key factor in development of oxidative stress in atherosclerotic lesions^[^^24^^]^. Based on our findings, the oxidative index of heart tissue, MDA content, was higher in diabetic rats compared to the normal rats (Fig. 2). A similar result has been reported by another investigation^[^^25^^]^. Since the high blood glucose is one of the most important factors that induce tissue oxidative stress, the positive feedback cycle between LOX-1 and oxygen free radicals is considered as important factor in atherosclerosis formation. Finally, it has been observed that atherosclerotic plaque rupturing correlates with overexpression of LOX-1 receptor^[^^26^^]^. These findings demonstrate the involvement of this protein in destabilization of atherosclerotic plaques heart attack. Thus, reducing LOX-1 expression may be a useful way in reducing diabetic complications such as atherosclerosis and other CVDs. 

Experimental and clinical studies have demonstrated some beneficial effects of exercise on coronary heart diseases^[^^11^^,^^27^^]^. Boule *et al*.^[^^28^^]^ in a meta-analysis study showed that aerobic training in diabetic patients decreased the risk of diabetic complications in comparison with normal people. In another meta-analysis study by Snowling and Hopkins^[^^29^^]^, they concluded that all modes of exercise training have small to moderate beneficial effects on glucose control in diabetic patients. However, in the current study, the swimming exercise did not significantly decrease the blood glucose of normal and diabetic rats (Table 1). It is possible that the type or intensity of exercise is an important factor for reduction of blood glucose during hyperglycemic condition. On the other hand, it has been shown that regular exercise decreases tissue oxidative stress^[^^30^^]^. Our results indicated that eight-week regular swimming exercise attenuated the parameters of oxidative and nitrosative stress of heart tissue (Figs. 2 and 3). These results are in agreement with the findings of other research. In one study, the regular treadmill running for eight weeks reduced the age-related increase of ROS production in liver of old rats^[^^31^^]^. In another study by Krause *et al.*^[^^32^^]^, it has been shown the reduction of muscular NOx by moderate-intensity exercise both in normal and diabetic situations. Therefore, there is a positive correlation between the level of physical activity and induction of antioxidant defense system in biological system^[^^33^^]^. Although hyperglycemia causes oxidative and nitrosative stress by excessive production of free radicals and impairment of antioxidant defense capacities^[^^19^^]^, based on our findings, we can conclude that eight weeks of regular swimming exercise is able to decrease the oxidative and nitrosative stress in heart (Figs. 2 and 3). It appears that this reduction happened probably by strengthening the antioxidant defense system of heart tissue. Also, according to our findings, we showed swimming exercise for eight weeks decreases the LOX-1 expression level in diabetic conditions (Fig. 1). The LOX-1 receptor stimulates the NADPH-oxidase enzyme, which produces oxygen free radicals^[^^22^^]^. Since this enzyme is the origin of oxidative stress in tissue and cell, it appears that the reduction of heart oxidative stress happens probably by reduction of LOX-1 receptor. Therefore, lowering LOX-1 receptor will be useful in prevention of atherosclerosis and other CVDs during diabetic conditions. 

Our findings indicated that swimming exercise is able to reduce heart expression of LOX-1 receptor, which overexpress during hyperglycemic conditions. This type of exercise also reduces different free radicals production of heart tissue possibly via reduction of LOX-1 receptor expression.
